# MMPs and TIMPs levels are correlated with anthropometric parameters, blood pressure, and endothelial function in obesity

**DOI:** 10.1038/s41598-021-99577-2

**Published:** 2021-10-08

**Authors:** Soumaya Boumiza, Karim Chahed, Zouhair Tabka, Marie-Paule Jacob, Xavier Norel, Gulsev Ozen

**Affiliations:** 1 Faculty of Medicine of Sousse, Department of Physiology and Functional Exploration, University of Sousse, UR 12ES06 Sousse, Tunisia; 2 Faculty of Sciences of Sfax, University of Sfax, Sfax, Tunisia; 3 INSERM U1148, LVTS, Eicosanoids and Vascular Pharmacology Group, CHU X. Bichat, 46 rue Huchard, 75018 Paris, France; 4 University of Sorbonne Paris North, 93430 Villetaneuse, France; 5 University of Paris, Paris, France; 6 Department of Pharmacology, Faculty of Pharmacy, Istanbul University, Istanbul, 34116 Turkey

**Keywords:** Biochemistry, Biomarkers, Cardiology, Diseases, Endocrinology, Medical research, Pathogenesis, Risk factors

## Abstract

The association between matrix metalloproteinases (MMPs), tissue inhibitor of metalloproteinases (TIMPs) and obesity as well as obesity-related disease including metabolic syndrome is not fully explored. Our aims are that: (i) to evaluate the plasma levels of MMP-1, MMP-2, MMP-3, MMP-9, TIMP-1, TIMP-2 and their ratios in non-obese people, overweight and obese people with or without metabolic syndrome, (ii) to investigate correlations between MMPs or TIMPs levels and several anthropometric parameters, blood pressure, endothelial function. Anthropometric and biochemical parameters were determined in 479 randomly selected participants, subdividing according to body mass index (BMI) and metabolic syndrome status. Plasma MMPs and TIMPs levels were measured. The assessment of endothelial function was characterized in people with obesity, overweight and non-obese, using laser Doppler Flowmetry. Obese people have elevated MMP-1, MMP-2, TIMP-1, TIMP-2 levels and decreased MMP-3/TIMP-1 and MMP-9/TIMP-1 ratios compared with non-obese people. MMP-1 levels and MMP-1/TIMP-1 ratio were positively correlated with BMI and waist circumference (WC) while MMP-2 levels were negatively correlated with BMI and WC values in obese people. MMP-3 levels and MMP-3/TIMP-1 ratio were positively correlated with systolic blood pressure (SBP) or diastolic blood pressure (DBP) in obese and metabolic syndrome people. Additionally, MMP-9 levels and MMP-9/TIMP-1 ratio were negatively correlated with endothelium-dependent response in obese and metabolic syndrome people. MMP-1, MMP-2, TIMP-1, TIMP-2 levels were increased in obese subjects. Significant correlations between anthropometric parameters and MMP-1 as well as MMP-1/TIMP-1 ratio supported these results. MMP-3 and -9 levels as well as their ratios with TIMP-1 were associated with blood pressure and endothelial-dependent response, respectively. In conclusion, our results demonstrated that MMP-1, MMP-3 and MMP-9 levels were correlated with several obesity-related parameters including BMI, WC, blood pressure and endothelial-dependent response. Our findings will hopefully provide new aspects for the use of MMPs and TIMPs as clinical biomarkers in obesity-related cardiovascular diseases such as metabolic syndrome and hypertension. The lack of measure of MMPs activity in plasma and relevant organs/tissues in obesity and metabolic syndrome is considered as a limitation in this report.

## Introduction

Obesity is a worldwide health problem with increasing incidence. This chronic metabolic disease is consistently and strongly associated with a higher risk of cardiovascular disease and mortality^1^. Furthermore, obesity leads to the risk-clustering status known as metabolic syndrome, which is characterized by the presence of any three of five factors including high waist circumference (WC), high plasma triglycerides (TG), low plasma high-density lipoprotein cholesterol (HDL-C), high fasting plasma glucose (FG), and high blood pressure^2^.

Matrix metalloproteinases (MMPs) are involved in physiological and pathological complications of obesity or metabolic syndrome through the degradation and remodeling of the extracellular matrix (ECM) molecules^3–5^. MMP family members are categorized into soluble collagenases (MMP-1, -8, -13), gelatinases (MMP-2, -9), stromelysins (MMP-3, -10, -11), matrilysins (MMP-7, -26), membrane-type MMPs (MT-MMPs) (MMP-14, -15, -16, -17, -24, -25) and elastase (MMP-12)^3^.

MMPs activity is regulated by their endogenous inhibitors tissue inhibitor of metalloproteinases (TIMPs): TIMP-1 inhibits MMP-1, MMP-3, MMP-7 and MMP-9, TIMP-2 inhibits especially MMP-2, TIMP-3 can inhibit MMP-2 and MMP-9, while TIMP-4 inhibits MT-1 MMP and MMP-2 activity^6^. Among these TIMPs, especially TIMP-1 and TIMP-2 gain importance in obesity-related cardiovascular diseases^7^. The balance between MMPs and TIMPs is a critical determinant of ECM integrity and function, and alterations in MMPs/TIMPs mediated proteolysis may contribute to many pathological states. Since TIMPs levels directly affect MMPs activity, changes of TIMPs levels play an important role in pathological conditions.

Several studies demonstrated altered expression of MMPs and TIMPs in obese rodent models^8–11^. The potential contribution of MMPs and TIMPs was also detected in mice with genetic deletion of MMPs or TIMPs^12,13^. In addition, mice treated with a global MMP inhibitor reduced adipose tissue weight and the total number of adipocytes when exposed to a high-fat diet^14^. The role of MMPs and TIMPs were also investigated in obesity-associated cardiovascular diseases including hypertension and metabolic syndrome^6,15–17^. Even though, several animal models demonstrated that there is a dysregulation of MMPs and TIMPs in these diseases, correlation between MMPs or TIMPs and blood pressure, several anthropometric parameters including body mass index, waist circumference is not fully examined in humans.

As well as altered MMPs, TIMPs levels, endothelial dysfunction has also been observed in obesity and metabolic syndrome^18,19^. Several in vivo approaches including assessment of forearm blood flow have been developed to measure the function of the endothelium in humans^19^. However, no previous work has studied the relationship between MMPs or TIMPs levels and endothelial dysfunction. Understanding this relationship could provide early treatment and prevention of more serious cardiovascular diseases such as atherosclerosis.

The aim of this study is to investigate the plasma levels of MMP-1, MMP-2, MMP-3, MMP-9, TIMP-1, TIMP-2 and their ratios in a large variety of study groups including overweight people and obese people with or without metabolic syndrome and non-obese healthy people with total of 479 participants. Furthermore, correlations between MMPs or TIMPs levels and endothelial function, several anthropometric parameters, blood pressure were performed.

## Materials and methods

### Study subjects

In the present study, we recruited 479 randomly selected Tunisian subjects, included 227 healthy lean (non-obese) people with BMI between 18.5 and 25 kg/m^2^, 67 overweight people with 25 ≤ BMI < 30 kg/m^2^, and 185 obese people with BMI ≥ 30 kg/m^2^. We followed the diagnostic criteria for metabolic syndrome defined according to the National Cholesterol Education Program's-Third Adult Treatment Panel (NCEP-ATPIII) which included the following factors: (1) increased WC (> 102 cm in men and > 88 cm in women); (2) elevated TG (≥ 150 mg/dL); (3) reduced HDL-C (< 1.03 mmol/L in men and < 1.29 mmol/L in women); (4) elevated blood pressure (≥ 130 mm Hg for SBP or ≥ 85 mm Hg for DBP); (5) elevated fasting glucose (FG) ≥ 6.1 mmol/L^20^. The presence of defined abnormalities in presence of three or more of the measures constitutes a diagnosis of the metabolic syndrome.

Depending on metabolic syndrome status, people with obesity were divided into two groups: 87 metabolic syndrome obese (MetsO) and 77 metabolic healthy obese (MHO). The study was approved by Farhat Hached Hospital Ethics Committee for research on humans in Tunisia. All research was performed in accordance with relevant guidelines/regulations, and informed consent was obtained from all participants and/or their legal guardians. This study has been performed in accordance with the Declaration of Helsinki. Information on each participant*’*s lifestyle and health status was obtained through an interview, including questions regarding smoking history, prescription medicines for diseases such as dyslipidemia, hypertension or cardiovascular diseases. Subjects with a history of cardiovascular, liver, renal, or thyroid disease, smoking habit, malignancy, diseases responsible for microvasculopathy and participants using medications that might affect lipid and glucose metabolism or alter the endothelial or smooth muscle dependent responses were excluded from the study. Other exclusion criteria were women in the menstrual cycle and pregnant women.

### Anthropometric measurements, biochemical analyses, and microvascular reactivity assessment

Participants were first examined anthropometrically. Height (m) and weight (kg) were taken with participants dressed in lightweight clothing without shoes and BMI was calculated (kg/m^2^). The WC was measured at the midway point between the lower rib margin and the crest of the ileum in a horizontal plane at standing position (cm), and the hip circumference (HC) was measured by placing a tape measure around the patient's hips at the level of the prominences over the greater trochanters of both femurs (cm); after, we calculated the waist-to-hip ratio (WHR) and the waist-to-height ratio (WHtR). Blood pressure was also measured using a mercury sphygmomanometer with an appropriately sized cuff and stethoscope after the subject has been seated for 5 min.

Blood samples were collected from subjects in tubes after 12 h overnight fast, the blood was maintained at 4 °C then centrifuged (4000*g* for 10 min). Plasma was distributed in aliquots and stored at − 80 °C until the batched measurements of parameters. FG was measured by the glucose oxidase method (Biomaghreb, Tunisia). Total cholesterol (TC) and TG were determined by the cholesterol oxidase and the glycerol oxidase methods (Elitech Diagnostic, France). HDL-C was measured by the immune-inhibition method (Elitech Diagnostic, France) and low-density lipoprotein cholesterol (LDL-C) concentrations were calculated with the Friedwald formula^21^. High-sensitivity C-reactive protein (hsCRP) concentrations were also measured. All biochemical parameters were determined on an automated Synchron CX7 Clinical System (Beckman, Fullerton, CA).

### Microvascular reactivity and endothelial function assessments

Microvascular reactivity parameters were determined as previously described^22^. Endothelial function was explored by assessing the forearm microvascular cutaneous vasoreactivity using laser Doppler flowmeter coupled with iontophoresis (Periflux System 5000, Perimed, Jarfalla Sweden). Endothelium-dependent vasodilation was evaluated by stimulation with 2% acetylcholine chloride (ACh) (Sigma Aldrich, Switzerland). Briefly, cutaneous blood flow was recorded at rest for 2 min and during the functional exploration. Three doses of ACh were delivered using an anodal current (0.1 mA for 10 s) at 2-min intervals. Data were expressed as cutaneous vascular conductance (CVC), which represents the ratio between the cutaneous blood flow and mean arterial pressure values, to take into account variations in blood pressure between subjects. The endothelium-dependent response was calculated as the difference between the peak CVC upon ACh stimulation (i.e., the CVC after the third dose of ACh) and the baseline CVC (ΔACh-CVC).

### BioPlex luminex and enzyme-linked immunosorbent assays

Firstly, as a preliminary study 9 MMPs (-1, -2, -3, -7, -8, -9, -10, -12, -13), TIMP-1, and TIMP-2 plasma levels were measured in non-obese and people with overweight and obesity by BioPlex Luminex (BioRad; 2 lasers, 3 colors, High-Throughput System for multiplex cytometric bead arrays). From the preliminary results given by this technique we have chosen to focus on MMP (-1, -2, -3 and -9) and TIMP-1 and TIMP-2. Then, MMP (-1, -2, -3 and -9) and TIMP-1 and TIMP-2 levels were determined by Enzyme-Linked Immunosorbent Assays (ELISA) using commercially available kits, human MMP-1 (DYS901), human MMP-2 (DY902), human MMP-3 (DY513), human MMP-9 (DY911), human TIMP-1 (DY970) and human TIMP-2 (DY971) (DUOSET®ELISA, R&D systems), respectively, according to the manufacturer's instructions.

### Statistical analysis

SPSS® 20.0 software (SPSS Inc., Chicago, IL, USA) and Graph Pad 7.0 software (La Jolla, CA, USA) were used for statistical analysis. The comparison of non-parametric quantitative variables was performed by the Mann Whitney test or the Kruskal–Wallis test. P values were adjusted for age, gender, systolic blood pressure, LDL-C, TC and FG (Table 2). The correlation study was carried out using the Spearman correlation coefficient. The β coefficient was determined by linear regression and binary logistic regression. A p-value less than 0.05 is considered significant.

### Ethical approval and informed consent

This study was approved by the local Ethical Committee of Farhat Hached Hospital (Sousse, Tunisia).

## Results

### Characteristics of the study population

The demographic and clinical characteristics of the participants are summarized in Table 1. Non-obese, overweight and obese groups had comparable gender distribution. However, obese and overweight people were older than non-obese people (P < 0.001 and 0.004, respectively; Table 1). In order to avoid the contribution of age, our results were adjusted for age. As expected, BMI and central obesity markers (WC, WHR, and WHtR) were higher in both overweight and obese people when compared to non-obese people (P < 0.001 for all; Table 1). Moreover, BMI, WC, WHR, and WHtR were significantly higher in people with obesity when compared to overweight people (Table 1). DBP was significantly increased in both overweight and obese people when compared to non-obese people (P < 0.001 for both groups, Table 1). Besides, SBP was significantly increased in obese when compared to non-obese people (P < 0.001, Table 1).

**Table 1:** Anthropometric, biochemical parameters and microvascular function of the study subjects according to obesity status.

Parameters	Non-obese (n = 227) (18.5 ≤ BMI < 25)	Overweight (n = 67) (25 ≤ BMI < 30)	Obese (n = 185) (BMI ≥ 30)	P^ϛ^ value	MHO (n = 87)	MetsO (n = 77)	P^ϛϛ^ value
**Demographic parameters**							
Gender (males/females)	101/126	34/33	92/93	0.42	37/40	48/39	0.23
Age (years)	34.81 ± 0.73	39.52 ± 1.53**	41.79 ± 0.81***	0.000	40.38 ± 1.30	43.10 ± 1.13	0.000
**Anthropometric parameters**							
BMI (kg/m^2^)	22.87 ± 0.13	28.12 ± 0.12***	37.69 ± 0.50***^###^	0.000	35.93 ± 0.70	38.52 ± 0.78^§§^	0.000
WC (cm)	79.93 ± 0.71	97.64 ± 1.05***	115.63 ± 1.06***^###^	0.000	110.64 ± 1.51	118.98 ± 1.48^§§§^	0.000
HC (cm)	95.07 ± 0.72	107.64 ± 0.91***	123.31 ± 1.12***^###^	0.000	120.79 ± 1.68	123.88 ± 1.63	0.000
WHR	0.84 ± 0.08	0.91 ± 0.01***	0.93 ± 0.006***^###^	0.000	0.91 ± 0.01	0.96 ± 0.008^§§^	0.000
WHtR	0.47 ± 0.004	0.57 ± 0.006***	0.70 ± 0.006***^###^	0.000	0.67 ± 0.01	0.72 ± 0.009^§§^	0.000
SBP (mmHg)	116.2 ± 0.6	122.8 ± 3.0	126.4 ± 0.13***	0.000	119.1 ± 1.6	133.0 ± 1.8 ^§§§^	0.000
DBP (mmHg)	76.7 ± 0.4	81.3 ± 1.5***	81.8 ± 0.07***	0.000	77.5 ± 1.0	85.3 ± 1.0 ^§§§^	0.000
**Biochemical parameters**							
hs-CRP (mg/dl)	1.28 ± 0.18	6.36 ± 1.01***	5.79 ± 0.42***	0.000	5.61 ± 0.6	5.90 ± 0.64	0.000
FG (mmol/l)	5.18 ± 0.11	5.32 ± 0.17	5.91 ± 0.12***^###^	0.000	5.30 ± 0.12	6.46 ± 0.19^§§§^	0.000
TG (mmol/l)	1.14 ± 0.05	1.55 ± 0.16***	1.62 ± 0.06***	0.000	1.57 ± 0.08	1.51 ± 0.11	0.000
TC (mmol/l)	4.45 ± 0.08	4.73 ± 0.13	4.98 ± 0.07***^#^	0.000	4.92 ± 0.10	5.15 ± 0.11	0.000
HDL-C (mmol/l)	1.26 ± 0.03	1.21 ± 0.04	1.11 ± 0.02***	0.000	1.26 ± 0.04	1.01 ± 0.02^§§§^	0.000
LDL-C (mmol/l)	2.67 ± 0.07	2.83 ± 0.11	3.16 ± 0.07***^#^	0.000	3.10 ± 0.11	3.25 ± 0.11	0.000
LDL-C/HDL-C_ratio_	2.37 ± 0.07	2.54 ± 0.15	3.05 ± 0.11***^#^	0.000	2.68 ± 0.15	3.37 ± 0.17^§§^	0.000
TC/HDL-C_ratio_	3.76 ± 0.10	4.11 ± 0.17	4.63 ± 0.13***^#^	0.000	4.03 ± 0.16	5.17 ± 0.20^§§§^	0.000
Microvascular function	Non-obese (n = 35) (18.5 ≤ BMI < 25)	Overweight (n = 13) (25 ≤ BMI < 30)	Obese (n = 38) (BMI ≥ 30)	P^ϛ^ value	MHO (n = 17)	MetsO (n = 19)	P^ϛϛ^ value
Basal CVC (PU/mm Hg)	0.09 ± 0.01	0.08 ± 0.02	0.056 ± 0.003**	0.01	0.06 ± 0.005	0.05 ± 0.004^§^	0.01
Peak ACh-CVC (PU/ mm Hg)	0.48 ± 0.04	0.43 ± 0.08	0.3 ± 0.03**	0.02	0.37 ± 0.05	0.27 ± 0.04^§^	0.03

Data are presented as mean ± SEM. P^ϛ^ indicates statistical differences intergroups (non-obese, overweight and obese subjects). P^ϛϛ^ indicates statistical differences intergroups (non-obese, MHO and MetsO). *P < 0.05 vs non-obese. **P < 0.01 vs non-obese. ***P < 0.001 vs non-obese. ^#^P < 0.05 vs overweight. ^###^P < 0.001 vs overweight. ^§^P < 0.05 vs MHO. ^§§^P < 0.01 vs MHO. ^§§§^P < 0.001 vs MHO.

*Ach* acetylcholine, *ApoA1* apolipoprotein A1, *ApoB* apolipoprotein B, *BMI* body mass index, *CVC* Cutaneous vascular conductance, *DBP* diastolic blood pressure, *FG* fasting glucose, *HC* hip circumference, *HDL-C* high density lipoprotein cholesterol, *hsCRP* high sensitivity C reactive protein, *LDL-C* low density lipoprotein cholesterol, *MetsO* Metabolic syndrome obese, *MHO* Metabolic healthy obese, *SBP* systolic blood pressure, *TC* total cholesterol, *TG* triglycerides, *WC* waist circumference, *WHR* waist to hip ratio, *WHtR* waist to height ratio. The sum does not add up to the total (MetsO/MHO) because of a few missing criteria in obese people.

Additionally, hs-CRP levels were significantly increased among obese and overweight people when compared to non-obese people (P < 0.001 for both groups, Table 1). FG levels were significantly increased in people with obesity when compared to non-obese and overweight people (P < 0.001, Table 1). As shown in Table 1, TG was significantly increased in both obese and overweight people when compared to non-obese people (P < 0.001 and < 0.001, respectively). The other lipid parameters (TC, LDL-C, LDL-C/HDL-C, and TC/HDL-C) were significantly increased in people with obesity when compared to overweight people (P < 0.05, for all; Table 1) and non-obese people (P < 0.001, for all; Table 1). On the contrary, HDL-C was significantly decreased in people with obesity when compared to non-obese people (P < 0.001; Table 1).

In our study, we have also categorized obese people according to metabolic status (MHO/MetsO). As shown in Table 1, non-obese, MHO, and MetsO groups had comparable gender distribution. No significant difference was found in the age when the MetsO and the MHO group were compared (P > 0.05, Table 1). Higher BMI values were found among MetsO when compared to MHO (P = 0.006, Table 1). Moreover, central obesity markers (WC, WHR, and WHtR) were significantly higher among MetsO when compared to MHO (P < 0.001, = 0.002 and 0.003 respectively, Table 1). SBP, DBP, and FG levels were significantly higher in MetsO than in MHO (P < 0.001 for all; Table 1). Lipid parameters TC/HDL-C and LDL-C/HDL-C were also significantly increased among MetsO when compared to MHO (P < 0.001 and 0.003, respectively; Table 1), while HDL-C was significantly decreased among MetsO when compared to MHO (P < 0.001, Table 1).

The association of microvascular endothelial function with obesity status was also performed. Basal CVC as well as peak ACh-CVC values were decreased in people with obesity when compared to non-obese people (p = 0.003 and = 0.005, respectively; Table 1). When subjects were categorized as metabolic syndrome status, we found a significant decrease in both basal CVC and peak ACh-CVC among MetsO group when compared to non-obese people (p = 0.012 and = 0.015, respectively; Table 1).

### Plasma levels of MMPs and TIMPs among study subjects

As shown in Table 2, plasma MMP-1 levels were significantly higher in the obese group than non-obese group (P = 0.015). Additionally, the levels of MMP-2 were markedly higher in the overweight and obese group than non-obese group (P < 0.001; P = 0.045, respectively, Table 2). In contrast, no significant difference was found in MMP-3 and MMP-9 levels when compared to the non-obese, overweight and obese groups (P = 0.15; P = 0.13, respectively, Table 2). Significantly increased levels of TIMP-1 were found in people with obesity when compared to non-obese people (P < 0.001, Table 2). In addition, a significant difference was found in TIMP-1 levels in obese people when compared to people with overweight (P = 0.008, Table 2). Similarly, the plasma concentration of TIMP-2 was significantly higher in the obese and overweight group than non-obese group (P = 0.008 and P = 0.006, respectively; Table 2).

**Table 2:** MMPs and TIMPs levels of the study population.

Parameters	Non-obese (18.5 ≤ BMI < 25)	Overweight (25 ≤ BMI < 30)	Obese (BMI ≥ 30)	MHO	MetsO
**MMPs**					
MMP-1 (ng/ml)	1.18 (0.04–9.97)	1.28 (0.08–5.8)	2.5 (0.08–22.5)*	2.7 (0.11–14.17)	1.84 (0.08–22.15)
MMP-2 (ng/ml)	100.65 (47.56–211.43)	117.67 (73.65–231.75)***	113.26 (43.98–260)*	116.77 (43.98–260)	110.46 (56.12–206)
MMP-3 (ng/ml)	6.22 (1.28–29.66)	9.42 (2.14–31.32)	7.46 (1.4–33)	7.95 (1.84–32.98)	7.91 (1.4–23.6)
MMP-9 (ng/ml)	63.02 (16.38–231.77)	54.19 (19.76–211.34)	66.28 (12.34–215.34)	67.2 (15.21–215.34)	65.72 (13.65–205.14)
**TIMPs**					
TIMP-1 (ng/ml)	141.05 (53.34–446.21)	173.28 (36.06–292.1)	193.32 (53.6–487.2)***^# #^	194.03 (53.62.76–413)	184.94 (70.54–487.22)
TIMP-2 (ng/ml)	90.26 (48.75–158.76)	103.72 (77.78–190.76)**	96.12 (65.93–268)**	97.99 (72.44–268)	94.47 (65.93–139.16) ^§^
**MMPs/TIMPs**					
MMP-1/TIMP-1	0.007 (0.003–0.01)	0.01 (0.005–0.01)	0.012 (0.006–0.02)	0.01 (0.007–0.02)	0.01 (0.005–0.02)
MMP-2/TIMP-2	1.11 (0.56–2.5)	1.11 (0.57–1.83)	1.15 (0.42–1.95)	1.12 (0.42–1.81)	1.19 (0.54–1.95)
MMP-3/TIMP-1	0.05 (0.02–0.09)	0.04 (0.02–0.09)	0.03 (0.02–0.07)***	0.04 (0.02–0.08)	0.04 (0.02–0.07)
MMP-9/TIMP-1	0.42 (0.09–8.61)	0.38 (0.03–2.09)	0.35 (0.05–1.69)*	0.34 (0.05–1.30)	0.39 (0.06–1.69)

Data are presented as median (interquartile range). Statistical differences were obtained with adjusted values for age, gender, systolic blood pressure, LDL-C, TC and FG. *P < 0.05 vs non-obese. **P < 0.01 vs non-obese. ***P < 0.001 vs non-obese. ^#^P < 0.05 vs overweight. ^##^P < 0.01 vs overweight. ^§^P < 0.05 MetsO vs MHO.

*MetsO* Metabolic syndrome obese, *MHO* Metabolic healthy obese, *MMP* matrix metalloproteinase, *TIMP* tissue inhibitors of MMP.

As shown in Table 2, no significant difference was found in MMP-1/TIMP-1 and MMP-2/TIMP-2 ratios when compared to the non-obese, overweight and obese groups (P > 0.05, for all; Table 2). In contrast, we had revealed a significant difference in MMP-9/TIMP-1 and MMP-3/TIMP-1 ratios when comparing the obese group with non-obese people (P = 0.04, P < 0.001, respectively; Table 2).

Subdividing the obese group into MetsO and MHO, a significant difference was found in TIMP-2 levels when comparing MetsO to MHO (P = 0.02, Table 2).

Binary logistic regression setting obesity and metabolic syndrome status as dependent variables and MMP (-1, -2, -3 and -9), TIMP (-1 and -2) and MMP/TIMP ratios (MMP-1/TIMP-1, MMP-2/TIMP-2, MMP-3/TIMP-1 and MMP-9/TIMP-1) as independent variables revealed a significant association of MMP-2, TIMP (-1 and -2) and MMP-2/TIMP-2 ratio when obesity status was considered as a dependent variable (Supplementary Table 1). However, no significant association was found when setting metabolic syndrome status as dependent variable (Supplementary Table 1). Bêta coefficient (β) for valences indicates the direction of the relationship between the predicted variable and the independent variables. In this regard, the probabilities of MMP-2, TIMP (-1 and -2) levels and MMP-2/TIMP-2 ratio have been increased in obese subjects (Supplementary Table 1).

### Spearman correlation of MMP-1 and MMP-2 plasma levels with anthropometric parameters (BMI and WC)

**Figure 1:**
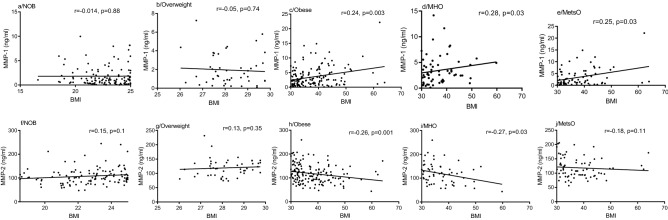
Spearman Rank correlation between BMI and MMP-1 and MMP-2 plasma levels respectively, according to obesity status. Regression coefficient (r) and statistical significance (p) are reported for each test. p-value < 0.05 is significant. *BMI* Body Mass Index, *NOB* non-obese, *MHO* metabolic healthy obese, *MetsO* metabolic syndrome obese.

**Figure 2:**
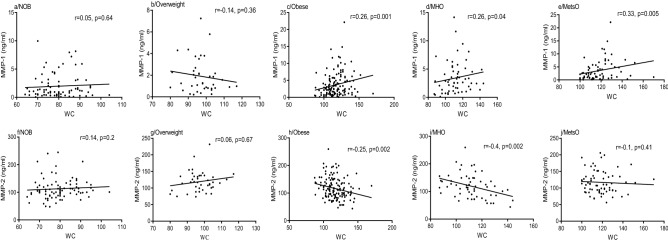
Spearman Rank correlation between WC and MMP-1 and MMP-2 plasma levels respectively, according to obesity status. Regression coefficient (r) and statistical significance (p) are reported for each test. p-value < 0.05 is significant. *NOB* non-obese, *MHO* metabolic healthy obese, *MetsO* metabolic syndrome obese, *WC* waist circumference.

MMP-1 levels and MMP-1/TIMP-1 ratio were positively correlated with BMI in obese people (r = 0.24, p = 0.003, Fig. 1c; r = 0.19, p = 0.016, Supplementary Table 2), however, no significant correlation was found among non-obese and overweight people (r = 0.014, p = 0.88; r = 0.05, p = 0.74, Fig. 1a and 1b). Subdividing people with obesity into MetsO and MHO, a positive correlation was also detected between MMP-1 and BMI values in both MetsO (r = 0.25, p = 0.03, Fig. 1e) and MHO (r = 0.28, p = 0.03, Fig. 1d). In addition, we found a positive correlation between MMP-1/TIMP-1 ratio and BMI in MetsO (r = 0.26, p = 0.023, Supplementary Table 2). Similarly, MMP-1 levels and MMP-1/TIMP-1 ratio were positively correlated with central adiposity (WC) in obese (r = 0.26, p = 0.001, Fig. 2c r = 0.18, p = 0.03, Supplementary Table 2), however, no significant correlation was noted among non-obese and overweight people (r = 0.05, p = 0.64; r = 0.14, p = 0.36, Fig. 2a,b, respectively). A positive correlation was also detected between MMP-1 levels and WC values in both MetsO (r = 0.33, p = 0.005, Fig. 2e) and MHO (r = 0.26, p = 0.04, Fig. 2d).

Conversely, MMP-2 levels were negatively correlated with BMI values among obese (r = − 0.26, p = 0.001, Fig. 1h) and MHO group (r = − 0.27, p = 0.03, Fig. 1i). However, no significant correlation was found among MetsO (r = − 0.18, p = 0.11; Fig. 1j), overweight (r = 0.13, p = 0.35, Fig. 1g) and non-obese people (r = 0.15, p = 0.1, Fig. 1f). MMP-2 levels were negatively correlated with WC values among obese (r = − 0.25, p = 0.002, Fig. 2h) and MHO (r = − 0.4, p = 0.002, Fig. 2i), however, no correlation was found among MetsO (r = − 0.10, p = 0,41, Fig. 2j), overweight (r = 0.06, p = 0.67, Fig. 2g) and non-obese people (r = 0.14, p = 0.2, Fig. 2f). MMP-3, MMP-9, TIMP-1 and TIMP-2 levels were not correlated with BMI or WC (Supplementary Table 2).

### Spearman correlation between MMP-3 plasma levels, SBP and DBP

**Figure 3:**
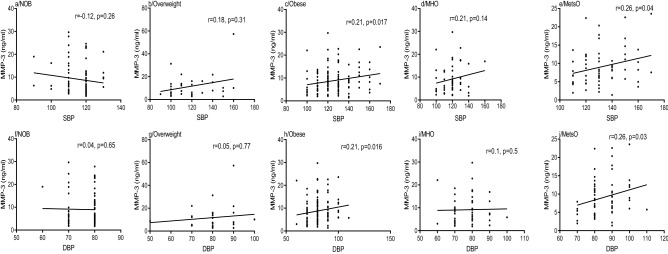
Spearman Rank correlation between MMP-3 plasma levels, SBP and DBP respectively, according to obesity status. Regression coefficient (r) and statistical significance (p) are reported for each test. p-value < 0.05 is significant. *DBP* diastolic blood pressure, *SBP* systolic blood pressure, *NOB* non-obese, *MHO* metabolic healthy obese, *MetsO* metabolic syndrome obese.

As shown in Fig. 3, MMP-3 levels were positively correlated with SBP in people with obesity (r = 0.21, p = 0.017, Fig. 3c), however, no significant correlation was noted among non-obese and overweight people (r = 0.12, p = 0.26; r = 0.18, p = 0.31, Fig. 3a,b, respectively).

Additionally, a positive correlation was found between MMP-3 levels and SBP in MetsO (r = 0.26, p = 0.04, Fig. 3e), however, no correlation was found among MHO (r = 0.21, p = 0.14, Fig. 3d). MMP-3 levels showed no correlation with DBP among MHO (r = 0.1, p = 0.5, Fig. 3i), overweight (r = 0.05, p = 0.77, Fig. 3g) and non-obese people (r = 0.04, p = 0.65, Fig. 3f). While, like MMP-3/TIMP-1 ratio (r = 0.29, p = 0.01, Supplementary Table 2), MMP-3 levels were positively correlated with DBP in MetsO (r = 0.26, p = 0.03, Fig. 3j) and obese people (r = 0.21, p = 0.016, Fig. 3h). In addition, TIMP-1 levels were negatively correlated with DBP among obese group (r = − 0.2, p = 0.027; Supplementary Table 2), however, no correlation was found among other groups (Supplementary Table 2).

### Spearman correlation between MMPs or TIMPs plasma levels and microvascular reactivity parameters (Basal CVC, Peak Ach CVC)

**Figure 4:**
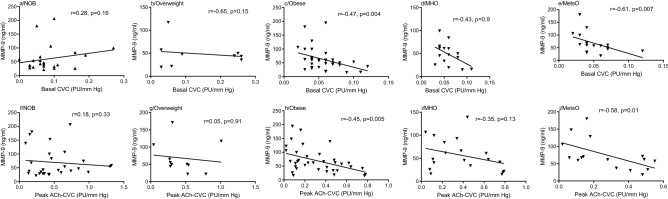
Spearman Rank correlation between MMP-9 plasma levels and microvascular parameters according to obesity status. Regression coefficient (r) and statistical significance (p) are reported for each test. p-value < 0.05 is significant. *Ach* acetylcholine, *CVC* cutaneous vascular conductance, *NOB* non-obese, *MHO* metabolic healthy obese, *MetsO* metabolic syndrome obese.

As shown in Fig. 4, MMP-9 levels were negatively correlated with basal CVC in people with obesity (r = − 0.47, p = 0.004, Fig. 4c), however, no correlation was found among non-obese and overweight people (r = 0.28, p = 0.16; r = 0.65, p = 0.15, Fig. 4a,b, respectively). Additionally, a negative correlation was found between MMP-9 levels and basal CVC in MetsO (r = − 0.61, p = 0.007, Fig. 4e), however, no correlation was found among MHO (r = − 0.43, p = 0.9, Fig. 4d). MMP-9 levels and MMP-9/TIMP-1 ratio showed no correlation with peak Ach-CVC among MHO (r = − 0.35, p = 0.13, Fig. 4i; r = − 0.24, p = 0.31, Supplementary Table 2), overweight (r = 0.05, p = 0.91, Fig. 4g; r = 0.08, p = 0.84, Supplementary Table 2) and non-obese people (r = 0.18, p = 0.33, Fig. 4f; r = 0.17, p = 0.34, Supplementary Table 2). While MMP-9 levels and MMP-9/TIMP-1 ratio were negatively correlated with peak Ach-CVC in obese (r = − 0.45, p = 0.005, Fig. 4h; r = − 0.46, p = 0.004, Supplementary Table 2) and MetsO subjects (r = − 0.58, p = 0.01, Fig. 4j; r = − 0.53, p = 0.03, Supplementary Table 2). In addition, MMP-2 levels and MMP-2/TIMP-2 ratio were positively correlated with Peak Ach-CVC among MetsO group (r = 0.55, p = 0.02; r = 0.6, p = 0.01; Supplementary Table 2), however, no correlation was found among other groups (Supplementary Table 2).

Linear regression analysis also identified that the MMP-9/TIMP-1 ratio is an independent variable affecting Peak-Ach-CVC in obese, MHO and MetsO subjects (Supplementary Table 3). Additionally, MMP-1, MMP-9, and MMP-1/TIMP-1 ratio were considered as independent variables affecting Peak Ach-CVC in MetsO subjects (Supplementary Table 3).

The Bêta coefficient (β) for valences indicates the direction of the relationship between the predicted variable and the independent variables. In this regard, the probabilities of impaired endothelial function in obese subjects increase when the parameters MMP-9, MMP-1, MMP-1/TIMP-1, and MMP-9/TIMP-1 ratios increase in obese or in MetsO subjects (Supplementary Table 3).

### Spearman correlation between MMPs or TIMPs plasma levels and CRP

MMP-1 and TIMP-1 levels were positively correlated with CRP in obese people (r = 0.21, p = 0.045; r = 0.2, p = 0.025, Supplementary Table 2), however, no significant correlation was found among non-obese and overweight people (Supplementary Table 2).

Subdividing people with obesity into MetsO and MHO, a negative correlation was also detected between MMP-2 levels, MMP-2/TIMP-2 ratio and CRP values in MetsO (r = − 0.26, p = 0.03, r = − 0.27, p = 0.03, respectively, Supplementary Table 2). However, no significant correlation was found among MHO subjects (Supplementary Table 2).

## Discussion

The main findings of the present study are that: (i) obese people have elevated MMP-1, MMP-2, TIMP-1, TIMP-2 levels and decreased MMP-3/TIMP-1, MMP-9/TIMP-1 ratios compared with non-obese people (ii) MMP-1 levels and MMP-1/TIMP-1 ratio were positively while MMP-2 levels were negatively correlated with BMI and WC values in people with obesity (iii) MMP-3 levels and MMP-3/TIMP-1 ratio were positively correlated with SBP and/or DBP in obese or metabolic syndrome people (iv) MMP-9 levels and MMP-9/TIMP-1 ratio were negatively correlated with the endothelium-dependent response in obese or metabolic syndrome people.

In the present study, we demonstrated higher circulating levels of MMP-1 in obese people as compared to non-obese people (Table 2). Since MMP-1 activity is regulated by TIMP-1, we have calculated MMP-1/TIMP-1 ratio. There was an increase of MMP-1/TIMP-1 ratio in obese people but this increase did not reach any significance (Table 2). There was no difference in MMP-1 levels and MMP-1/TIMP-1 ratio between obese people with or without metabolic syndrome (Table 2). The association of MMP-1 levels and obesity was also verified by significant positive correlations between MMP-1 levels or MMP-1/TIMP-1 ratio and BMI/WC values in people with obesity (Figs. 1c and 2c, Supplementary Table 2). It has been suggested that macrophage accumulation in adipose tissue play important role for the stimulation of MMP-1 production by preadipocytes, mediated by pro-inflammatory mediators^23^. Our results demonstrated that the levels of CRP, mediator of inflammation, is increased in obesity (Table 1). Inflammation observed in obesity could be stimulant of MMP-1 levels and could be involved in adipose tissue remodeling during adipose tissue expansion in obesity^23^. Positive correlation observed between MMP-1 and CRP levels supported this hypothesis (Supplementary Table 2). Similar results were obtained^24^ and discussed in our previous studies^22,24^. On the other hand, studies performed on relatively small numbers of participants (n = 54–102) showed that there is no difference in MMP-1 levels between obese and non-obese people^25,26^. It should be noted that our recent study indicated that MMP-1 levels were increased only in obese women, not in obese men^24^. This result suggested that sex hormones could participate in MMP-1 regulation in obesity. The contribution of MMP-1 on obesity development was also demonstrated in studies including ours showing an association between polymorphisms of the MMP-1 gene and BMI values^22,27^. Another study demonstrated an association of MMP-1 genotypes/ haplotypes with MMP-1 levels predominantly in non-obese subjects^28^. Despite the contradictory effect of MMP-1 on obesity, the majority of studies indicated increased levels of MMP-1 in obesity.

Our results exhibited that MMP-2 levels were markedly higher in obese or overweight subjects than non-obese people (Table 2). Interestingly, there is a small reduction in obese people versus overweight people even though it did not reach significance. This probably leads to a significant negative correlation between MMP-2 levels and BMI/WC values in people with obesity however this correlation was not observed in overweight or non-obese people (Fig. 1f–h and Fig. 2f–h, respectively). This result suggests that MMP-2 levels were increased in the development of obesity while in obese people MMP-2 levels were decreased gradually depending on obesity severity that could be a compensatory mechanism.

MMP-2 activity is regulated by TIMP-2. The interaction between TIMP-2 and pro-MMP-2 is also part of activation process of pro-MMP, because it allows the binding of the complex MMP-2/TIMP-2 a key step in the generation of the active form of MMP-2. At higher levels of TIMP-2, pro-MMP-2 activation is prevented^29,30^. Our results showed obese people with or without metabolic syndrome exhibited no difference in MMP-2 levels as well as MMP-2/TIMP-2 ratio (Table 2). Several studies demonstrated that plasma MMP-2 levels or MMP-2 gene expression in visceral adipose tissue were not modified in obese or metabolic syndrome people^31–35^. Furthermore, in morbidly obese women, serum MMP-2 concentration was not affected by significant weight loss after bariatric surgery^36^. On the other hand, some studies exhibited either increase or decrease in MMP-2 levels or activity in obese or metabolic syndrome^37–42^. Limitations of these studies were their small sample size which may affect the power of their analysis. Furthermore, it is possible that gender or methodological differences have affected the results. It is worth mentioning that our study included 479 participants and age, gender, systolic blood pressure, LDL-C, TC and FG adjustments have been performed.

In the present study, we demonstrated that there is no difference in circulating levels of MMP-3 between obese, overweight, and non-obese people (Table 2). Furthermore, no difference was found in obese people with and without metabolic syndrome (Table 2). In agreement with our results, there was no difference in MMP-3 levels of obese subjects with metabolic syndrome^34^. Furthermore, MMP-3 levels were not affected by significant weight loss people with obesity^31,36–40,43^. On the other hand, Traurig et al*.* demonstrated that MMP-3 levels were downregulated in preadipocytes/stromal vascular cells from obese subjects, and real-time PCR showed that MMP-3 expression levels are negatively correlated with percent body fat^44^. Similarly, genetic deletion of MMP-3 gene in mice resulted in increased body weight^8^. Another study demonstrated that, high fat diet decreased MMP-3 activity in female mice^45^. In line with these results, we have obtained decreased MMP-3/TIMP-1 ratio in obese people (Table 2).

Although obesity plays a key role in the development of hypertension, the mechanism underlying this effect has not been fully evaluated. Our results demonstrated a significant positive correlation between MMP-3 levels or MMP-3/TIMP-1 ratio and SBP/DBP in obese people (Fig. 3c,h). In line with our results, serum levels of MMP-3 were positively associated with arterial stiffness^46,47^ which is the primary cause of isolated systolic hypertension^48^. Moreover, the polymorphism of MMP-3 was associated with blood pressure and arterial stiffness^49,50^. Overall, MMP-3 gene variant seems to contribute to the development of hypertension by affecting arterial stiffness^51^. Another study group suggested that MMP-3 polymorphism could be beneficial in predicting risk for hypertension^52^.

Our results demonstrated that there is no difference in MMP-9 levels between obese, overweight, metabolic syndrome people, and non-obese people (Table 2). There are conflicting results regarding MMP-9 levels in obesity and metabolic syndrome. Several studies exhibited greater MMP-9 levels^34,37,38,53–55^ whereas others demonstrated either no difference^33,39^ or decreased MMP-9 levels in obese or metabolic syndrome^9^. In our results, we have found decreased MMP-9/TIMP-1 ratio in obese people (Table 2). There are several methodological discrepancies between studies, such as differences in gender distribution, age, species, and samples from which measurements were performed. Furthermore, several studies indicated a lack of association between adipose tissue and plasma levels of MMP-9, suggesting that this tissue is not a major contributor to circulating MMP-9 levels^56,57^.

Even though MMP-9 levels were not affected by obesity status, they are negatively correlated with endothelium-dependent response measured by forearm blood flow (Fig. 4). We have obtained similar results with MMP-9/TIMP-1 ratio (Supplementary Table 2). Endothelial dysfunction is one of the early determinants for obesity-related diseases including hypertension. Although the association between MMP-9 levels and hypertension was detected in several studies^7,58^, the mechanism underlying this effect was not investigated. Our results emphasized that MMP-9 levels or MMP-9/TIMP-1 ratio could be predictive for endothelial dysfunction. We suggest that more serious diseases related to endothelial dysfunction such as coronary artery disease could be prevented by measuring MMP-9 levels.

Our results exhibited that TIMP-1 levels were markedly and gradually increased in people with obesity as compared to overweight and non-obese people (Table 2). In line with our results, several studies demonstrated increased TIMP-1 levels either in adipose tissue or plasma-derived from people with obesity or experimental animals^9,10,26,37,59–62^. Compared with these studies, our results by stratifying obese population into different groups based on BMI and metabolic syndrome status further investigate TIMP-1 levels. Our results demonstrated that there is a progressive increase in TIMP-1 levels depending on obesity status. The increase in TIMP-1 levels in people with overweight could be an early predictor for obesity and obesity-related cardiovascular diseases. The role of TIMP-1 in obesity development was also supported by experimental studies. TIMP-1 increased the accumulation of lipid during differentiation of 3T3-L1 adipocytes^63^ and protection from obesity was observed in mice lacking TIMP-1 gene^64^. Similary, obese Zucker mice demonstrated increased TIMP-1 protein and activity^11^. On the other hand, there are some contradictory results regarding on TIMP-1 levels in obesity. Two studies performed on relatively low number of patients demonstrated that there was no change in TIMP-1 levels between obese and lean people^65^. Another study demonstrated that obesity was observed in mice with deletion of TIMP-1 gene^12,66^.

Our results exhibited that TIMP-2 levels were significantly increased in people with obesity and overweight compared to non-obese people (Table 2). In support of our findings, obese children and adolescents had greater TIMP-2 levels^33^. However, another study performed on premenopausal obese women exhibited no differences in TIMP-2 levels^37^. These conflicting results suggested a contribution of sex hormones in the regulation of TIMP-2 levels in obesity. Our results demonstrated that there was a significant difference in TIMP-2 levels between obese people with and without metabolic syndrome and greater levels of TIMP-2 in those groups versus non-obese subjects were observed. In accordance with our results, higher TIMP-2 levels were detected in patients with metabolic syndrome versus non-obese people^54,67^.

One of limitations is that MMPs activity is not investigated in our study. MMPs activity is also largely determined by MMPs/TIMPs balance and in this study we present our results as MMP/TIMP ratios. Since in literature there is a lack of information about MMPs activity in humans, the implication of MMPs activity in obesity, metabolic syndrome and endothelial dysfunction should be evaluated in future studies. Other limitation is that plasma levels of MMPs and TIMPs did not represent alterations in several organs and tissue including adipose tissue, heart or blood vessels compromised in obesity or metabolic syndrome. In our recent study we demonstrated that adipose tissue and blood vessels released large amount of MMPs and TIMPs which are regulated by inflammation^24^. Other study showed that perivascular adipose tissue derived from obese rats thoracic-aorta had increased MMP-9 activity, which was associated with increased stiffness^68^. Further studies to investigate MMPs activity in plasma and other organs and tissues in humans are necessary.

In conclusion, our results demonstrated that increased levels of MMP-1, MMP-2, TIMP-1, TIMP-2 and decreased ratios of MMP-3/TIMP-1 and MMP-9/TIMP-1 were detected in obese people while there was no difference in MMP-3 and MMP-9 levels. Significant correlations between anthropometric parameters and MMP-1 as well as MMP-2 levels supported these results. Furthermore, our results showing the correlations between MMP-3, -9, their ratios with TIMP-1 and blood pressure as well as endothelial-dependent response could be beneficial for the prevention of obesity-related cardiovascular diseases such as hypertension or atherosclerosis. Our findings will hopefully provide new aspects for the use of MMPs and TIMPs as clinical biomarkers in obesity-related cardiovascular diseases.

## Supplementary Information

Supplementary Information.
