# Supplementary material for: MMPs and TIMPs levels are correlated with anthropometric parameters, blood pressure, and endothelial function in obesity
Source: Sci Rep. 2021 Oct 8;11:20052. doi: 10.1038/s41598-021-99577-2 (PMC8501083; doi:10.1038/s41598-021-99577-2)
Supplement: Supplementary file 1 — Supplementary Information. [file 41598_2021_99577_MOESM1_ESM.docx]

**MMPs and TIMPs levels are correlated with anthropometric parameters, blood pressure, and endothelial function in obesity**

**Soumaya Boumiza,^1^ Karim Chahed ^1,2^, Zouhair Tabka^1^, Marie-Paule Jacob^3^, Xavier Norel^3,4,5^, Gulsev Ozen^6*^**

^1^Faculty of Medicine of Sousse, Department of Physiology and Functional Exploration, University of Sousse, UR 12ES06 Sousse, Tunisia

^2^ Faculty of Sciences of Sfax, University of Sfax, Sfax, Tunisia

^3^ INSERM U1148, LVTS, Eicosanoids and Vascular Pharmacology Group, CHU X. Bichat, 46 rue Huchard, 75018 Paris, France.

^4^ University of Sorbonne Paris North, 93430 Villetaneuse, France.

^5^ University of Paris, France.

^6^Department of Pharmacology, Faculty of Pharmacy, Istanbul University, Istanbul, Turkey

***Corresponding author:** Gulsev Ozen

Department of Pharmacology, Faculty of Pharmacy, Istanbul University, Istanbul 34116, Turkey.

e-mail: [gulsevozen@istanbul.edu.tr](mailto:gulsevozen@istanbul.edu.tr)

**Supplementary Table 1** Binary logistic regression analysis

| **Independent variables** | **Non obese-Obese subjects** | | **MHO-MetsO** | |
| --- | --- | --- | --- | --- |
|  | β coefficient | P-value | β coefficient |  |
| MMP-1 | 1.59 | 0.64 | 1.33 | 0.11 |
| MMP-2 | 1.06 | **0.008** | 0.99 | 0.91 |
| MMP-3 | 0.94 | 0.29 | 1.02 | 0.72 |
| MMP-9 | 0.99 | 0.61 | 0.98 | 0.32 |
| TIMP-1 | 1.01 | **0.01** | 0.99 | 0.56 |
| TIMP-2 | 0.95 | **0.04** | 0.98 | 0.65 |
| MMP-1/TIMP-1 | 0.99 | 0.61 | 0.02 | 0.11 |
| MMP-2/TIMP-2 | 0.008 | **0.03** | 2.15 | 0.84 |
| MMP-3/TIMP-1 | 0.99 | 0.36 | 0.014 | 0.7 |
| MMP-9/TIMP-1 | 1.46 | 0.77 | 7.01 | 0.33 |

Statistical significance (p) and β coefficient are reported for each test. p-value <0.05 is significant.

**Supplementary Table 2** Spearman Rank correlation between MMPs and TIMPs plasma levels, anthropometric parameters, biochemical analyses and microvascular function according to obesity status.

|  |  | Non-obese (r,P) | | | | | |
| --- | --- | --- | --- | --- | --- | --- | --- |
|  | CRP | BMI | WC | SBP | DBP | Basal CVC | Peak CVC-ACh |
| MMP-1 | 0.33, 0.86 |  |  | 0.09, 0.36 | 0.14, 0.17 | -0.09, 0.66 | -0.16, 0.4 |
| MMP-2 | -0.06, 0.57 |  |  | -0.13, 0.22 | 0.06, 0.58 | 0.41, 0.37 | 0.1, 0.6 |
| MMP-3 | 0.18, 0.09 | 0.15, 0.1 | 0.4, 0.25 |  |  | -0.035, 0.87 | 0.18, 0.33 |
| MMP-9 | 0.14, 0.18 | 0.12, 0.2 | 0.14, 0.2 | 0.19, 0.86 | 0.44, 0.2 |  |  |
| TIMP-1 | -0.002, 0.98 | -0.04, 0.1 | -0.4, 0.7 | 0.21, 0.26 | 0.04, 0.71 | -0.3, 0.12 | -0.4, 0.2 |
| TIMP-2 | -0.12, 0.25 | -0.06, 0.47 | 0.04, 0.7 | 0.1, 0.33 | -0.26, 0.12 | -0.16, 0.44 | -0.14, 0.44 |
| MMP-2/TIMP-2 | 0.01, 0.92 | **0.21, 0.02** | 0.06, 0.57 | 0.05, 0.58 | 0.18, 0.07 | **0.43, 0.03** | 0.07, 0.7 |
| MMP-9/TIMP-1 | 0.14, 0.17 | 0.035, 0.71 | -0.03, 0.77 | 0.03, 0.72 | **0.24, 0.02** | **0.72, P<0.001** | 0.17, 0.34 |
| MMP-1/TIMP-1 | -0.09, 0.36 | 0.05, 0.6 | 0.15, 0.18 | -0.07, 0.47 | 0.01, 0.93 | 0.22, 0.3 | 0.17, 0.38 |
| MMP-3/TIMP-1 | 0.14, 0.17 | 0.13, 0.15 | 0.14, 0.16 | -0.19, 0.07 | 0.05, 0.61 | 0.08, 0.7 | 0.06, 0.41 |
|  |  | Overweight (r,P) | | | | | |
|  | CRP | BMI | WC | SBP | DBP | Basal CVC | Peak CVC-ACh |
| MMP-1 | 0.02, 0.88 |  |  | 0.11, 0.55 | -0.003, 0.98 | -0.65, 0.15 | 0.44, 0.27 |
| MMP-2 | -0.05, 0.77 |  |  | -0.1, 0.56 | -0.05, 0.74 | -0.25, 0.63 | 0.4, 0.33 |
| MMP-3 | 0.16, 0.35 | 0.87, 0.63 | 0.22, 0.15 |  |  | 0.37, 0.46 | 0.13, 0.75 |
| MMP-9 | -0.07, 0.7 | 0.13, 0.36 | 0.05, 0.76 | 0.24, 0.18 | 0.08, 0.65 |  |  |
| TIMP-1 | -0.07, 0.67 | 0.07, 0.7 | -0.02, 0.9 | -0.05, 0.77 | -0.09, 0.6 | 0.08, 0.87 | -0.61, 0.1 |
| TIMP-2 | -0.02, 0.91 | -0.09, 0.5 | -0.03, 0.84 | -0.21, 0.23 | -0.04, 0.18 | 0.31, 0.54 | 0.24, 0.56 |
| MMP-2/TIMP-2 | -0.07, 0.65 | 0.17, 0.22 | 0.14, 0.35 | 0.1, 0.6 | -0.11, 0.51 | -0.3, 0.57 | -0.03, 0.93 |
| MMP-9/TIMP-1 | -0.01, 0.94 | 0.12, 0.41 | -0.11, 0.48 | -0.01, 0.94 | 0.03, 0.85 | -0.45, 0.37 | 0.08, 0.84 |
| MMP-1/TIMP-1 | 0.04, 0.81 | 0.11, 0.44 | -0.018, 0.91 | 0.33, 0.06 | 0.2, 0.26 | -0.62, 0.18 | 0.26, 0.52 |
| MMP-3/TIMP-1 | 0.21, 0.21 | 0.08, 0.57 | 0.16, 0.31 | 0.22, 0.21 | 0.11, 0.51 | -0.23, 0.65 | 0.55, 0.15 |
|  |  | Obese (r, P) | | | | | |
|  | CRP | BMI | WC | SBP | DBP | Basal CVC | Peak CVC-ACh |
| MMP-1 | **0.2, 0.045** |  |  | -0.04, 0.6 | -0.01, 0.91 | -0.005, 0.97 | -0.1, 0.6 |
| MMP-2 | -0.11, 0.18 |  |  | 0.036, 0.7 | -0.006, 0.94 | -0.07, 0.68 | 0.12, 0.47 |
| MMP-3 | -0.02, 0.78 | -0.1, 0.2 | 0.01, 0.9 |  |  | 0.08, 0.64 | -0.24, 0.14 |
| MMP-9 | 0.13, 0.12 | -0.008, 0.3 | -0.08, 0.3 | -0.08, 0.33 | -0.004, 0.96 |  |  |
| TIMP-1 | **0.2, 0.025** | 0.14, 0.1 | 0.1, 0.2 | -0.09, 0.31 | **-0.2, 0.027** | -0.15, 0.38 | 0.027, 0.87 |
| TIMP-2 | 0.08, 0.32 | -0.01, 0.22 | -0.07, 0.37 | 0.04, 0.64 | 0.06, 0.46 | 0.3, 0.08 | -0.05, 0.74 |
| MMP-2/TIMP-2 | -0.17, 0.06 | -0.13, 0.1 | -0.16, 0.06 | 0.03, 0.74 | 0.02, 0.81 | -0.2, 0.23 | 0.04, 0.78 |
| MMP-9/TIMP-1 | 0.04, 0.63 | -0.07, 0.37 | -0.03, 0.67 | 0.03, 0.67 | 0.13, 0.14 | -0.27, 0.11 | **-0.46, 0.004** |
| MMP-1/TIMP-1 | 0.11, 0.2 | **0.19, 0.016** | **0.18, 0.03** | 0.06, 0.51 | 0.03, 0.74 | 0.02, 0.87 | 0.07, 0.66 |
| MMP-3/TIMP-1 | -0.1, 0.22 | -0.15, 0.06 | -0.07, 0.41 | **0.22, 0.01** | 0.18, 0.37 | 0.06, 0.71 | -0.22, 0.18 |
|  |  | MHO (r, P) | | | | | |
|  | CRP | BMI | WC | SBP | DBP | Basal CVC | Peak CVC-ACh |
| MMP-1 | 0.08, 0.55 |  |  | 0.12, 0.4 | 0.32, 0.2 | 0.28, 0.3 | -0.03, 0.9 |
| MMP-2 | 0.01, 0.92 |  |  | -0.07, 0.62 | -0.07, 0.62 | -0.15, 0.57 | 0.04, 0.87 |
| MMP-3 | 0.03, 0.82 | 0.008, 0.94 | 0.013, 0.92 |  |  | -0.12, 0.65 | -0.52, 0.22 |
| MMP-9 | 0.18, 0.21 | -0.17, 0.18 | -0.2, 0.12 | 0.14, 0.3 | 0.07, 0.63 |  |  |
| TIMP-1 | 0.06, 0.64 | -0.04, 0.1 | -0.06, 0.6 | -0.004, 0.97 | 0.03, 0.81 | 0.03, 0.9 | 0.25, 0.3 |
| TIMP-2 | 0.21, 0.12 | 0.11, 0.37 | 0.05, 0.7 | 0.16, 0.24 | 0.03, 0.8 | -0.26, 0.33 | -0.002, 0.98 |
| MMP-2/TIMP-2 | -0.05, 0.7 | -0.27, 0.036 | -0.41, 0.001 | -0.11, 0.42 | -0.09, 0.53 | -0.27, 0.32 | -0.01, 0.95 |
| MMP-9/TIMP-1 | 0.15, 0.26 | -0.08, 0.54 | -0.12, 0.33 | 0.07, 0.6 | -0.1, 0.52 | -0.38, 0.17 | -0.24, 0.31 |
| MMP-1/TIMP-1 | 0.03, 0.8 | 0.1, 0.43 | 0.17, 0.2 | 0.14, 0.32 | 0.25, 0.07 | 0.3, 0.26 | 0.1, 0.98 |
| MMP-3/TIMP-1 | -0.006, 0.96 | -0.05, 0.7 | 0.03, 0.8 | 0.23, 0.1 | -0.05, 0.68 | -0.17, 0.53 | -0.33, 0.16 |
|  |  | MetsO(r, P) | | | | | |
|  | CRP | BMI | WC | SBP | DBP | Basal CVC | Peak CVC-ACh |
| MMP-1 | 0.17, 0.17 |  |  | 0.013, 0.92 | -0.11, 0.35 | -0.3, 0.24 | -0.3, 0.26 |
| MMP-2 | -**0.26, 0.03** |  |  | 0.1, 0.41 | 0.05, 0.68 | -0.1, 0.7 | 0.55, 0.02 |
| MMP-3 | -0.09, 0.47 | -0.05, 0.68 | -0.1, 0.38 |  |  | 0.46, 0.06 | -0.1, 0.73 |
| MMP-9 | 0.12, 0.31 | -0.02, 0.87 | -0.03, 0.8 | -0.11, 0.37 | -0.009, 0.94 |  |  |
| TIMP-1 | 0.19, 0.12 | 0.13, 0.24 | 0.14, 0.24 | -0.035, 0.78 | -0.25, 0.4 | -0.046, 0.07 | -0.07, 0.8 |
| TIMP-2 | -0.03, 0.77 | -0.18, 0.12 | -0.01, 0.93 | 0.2, 0.1 | 0.26, 0.35 | 0.42, 0.08 | 0.07, 0.78 |
| MMP-2/TIMP-2 | **-0.27, 0.03** | -0.001, 0.99 | -0.03, 0.8 | -0.007, 0.95 | -0.02, 0.84 | -0.27, 0.26 | **0.6, 0.01** |
| MMP-9/TIMP-1 | 0.03, 0.76 | 0.001, 0.99 | 0.06, 0.6 | -0.07, 0.56 | 0.16, 0.19 | -0.24, 0.33 | **-0.53, 0.03** |
| MMP-1/TIMP-1 | 0.15, 0.23 | **0.26, 0.023** | 0.23, 0.05 | 0.11, 0.41 | -0.05, 0.68 | -0.1, 0.68 | 0.01, 0.96 |
| MMP-3/TIMP-1 | -0.16, 0.21 | -0.14, 0.22 | -0.09, 0.42 | 0.2, 0.1 | **0.29, 0.01** | 0.46, 0.05 | -0.04, 0.88 |

Regression coefficient (r) and statistical significance (p) are reported for each test. p-value <0.05 is significant.

*Ach* acetylcholine, *BMI* body mass index, *CVC* cutaneous vascular conductance, *DBP* diastolic blood pressure,

*NOB* non-obese, *MHO* metabolic healthy obese, *MetsO* metabolic syndrome obese, *SBP* Systolic blood pressure.

**Supplementary Table 3** Linear regression analysis: independent factors related to Peak ACh-CVC in function of subgroups.

| **Parameters** | **Non- obese** |  | **Obese** |  | **MHO** |  | **MetsO** |  |
| --- | --- | --- | --- | --- | --- | --- | --- | --- |
| Peak ACh-CVC (dependant factor) | **Bêta** | **P** | **Bêta** | **P** | **Bêta** | **P** | **Bêta** | **P** |
| MMP-1 | -4.54 | 0.15 | -2.62 | 0.16 | -0.81 | 0.35 | **-9.29** | **0.022** |
| MMP-2 | -2.88 | 0.24 | 2.6 | 0.3 | -0.33 | 0.57 | 4.72 | 0.06 |
| MMP-3 | 4.45 | 0.45 | -2.01 | 0.16 | 7.02 | 0.31 | -0.35 | 0.43 |
| MMP-9 | -4.54 | 0.43 | 0.34 | -1.3 | -0.06 | 0.1 | -1.7 | **0.045** |
| TIMP-1 | 0.66 | 0.36 | 0.97 | 1.62 | 0.32 | 0.22 | -0.6 | 0.48 |
| TIMP-2 | 2.45 | 0.11 | -1.32 | 0.03 | 0.97 | 0.1 | -2.92 | 0.064 |
| MMP-1/TIMP-1 | 0.88 | 0.2 | 2.24 | 0.17 | 1.71 | 0.15 | -10.45 | **0.04** |
| MMP-2/TIMP-2 | 3.02 | 0.18 | -1.97 | 0.4 | 0.56 | 0.4 | -4.08 | 0.11 |
| MMP-3/TIMP-1 | -3.21 | 0.08 | 2.02 | 0.23 | -2.95 | 0.08 | -1.44 | 0.18 |
| MMP-9/TIMP-1 | 4.02 | 0.15 | -0.98 | **0.025** | -1.62 | **0.041** | -3.57 | **0.028** |

Statistical significance (p) and β coefficient are reported for each test. p-value <0.05 is significant. *Ach* acetylcholine, *CVC* cutaneous vascular conductance.
